# 

**Published:** 1892-07-30

**Authors:** 


Per Annum, 8s. 6d., post free.

Monthly Parts, 6d.; post free, 8d.

Ditto per Annum, 8s., post free.

### THE HOSPITAL

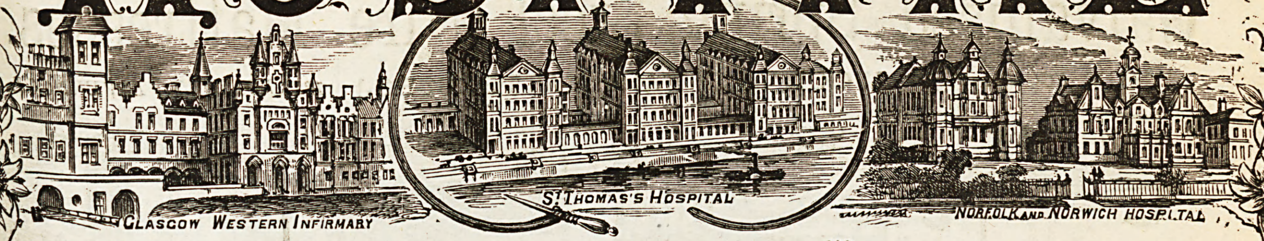

WITH EXTRA NURSING SUPPLEMENT.

No. 305. Vol. XII.] SATURDAY, JULY 30th, 1892. [TWOPENCE.

